# Encoding speech rate in challenging listening conditions: White noise and reverberation

**DOI:** 10.3758/s13414-022-02554-8

**Published:** 2022-08-22

**Authors:** Eva Reinisch, Hans Rutger Bosker

**Affiliations:** 1 Acoustics Research Institute, Austrian Academy of Sciences, Wohllebengasse 12-14, 1040 Vienna, Austria; 2 Max Planck Institute for Psycholinguistics, PO Box 310, 6500 AH Nijmegen, The Netherlands; 3 Donders Institute for Brain, Cognition and Behaviour, Radboud University, Nijmegen, The Netherlands

**Keywords:** Speech rate, Degraded speech perception, Psycholinguistics, Spoken word recognition

## Abstract

Temporal contrasts in speech are perceived relative to the speech rate of the surrounding context. That is, following a fast context sentence, listeners interpret a given target sound as longer than following a slow context, and vice versa. This rate effect, often referred to as “rate-dependent speech perception,” has been suggested to be the result of a robust, low-level perceptual process, typically examined in quiet laboratory settings. However, speech perception often occurs in more challenging listening conditions. Therefore, we asked whether rate-dependent perception would be (partially) compromised by signal degradation relative to a clear listening condition. Specifically, we tested effects of white noise and reverberation, with the latter specifically distorting temporal information. We hypothesized that signal degradation would reduce the precision of encoding the speech rate in the context and thereby reduce the rate effect relative to a clear context. This prediction was borne out for both types of degradation in Experiment 1, where the context sentences but not the subsequent target words were degraded. However, in Experiment 2, which compared rate effects when contexts and targets were coherent in terms of signal quality, no reduction of the rate effect was found. This suggests that, when confronted with coherently degraded signals, listeners adapt to challenging listening situations, eliminating the difference between rate-dependent perception in clear and degraded conditions. Overall, the present study contributes towards understanding the consequences of different types of listening environments on the functioning of low-level perceptual processes that listeners use during speech perception.

## Introduction

Speech is a highly variable acoustic signal that listeners have to map onto their language system (e.g., words) in order to understand what is being said. Moreover, the listening environment is hardly ever quiet, but rather the to-be-decoded speech signal may be heard in background noise or distorted by room acoustics. Nevertheless, intuitively, speech perception does not seem like a major challenge to most listeners. This is because the human brain has a number of processes at its disposal that help listeners deal with the variability in the signal. The present study focuses on one of these processes— namely, rate-dependent speech perception—whereby listeners use earlier temporal information—that is, speech rate—in a preceding context sentence to recognize upcoming words. Specifically, we test how robustly the temporal information in a preceding context sentence is encoded in challenging listening conditions, such as background noise and reverberation.

Rate-dependent perception is typically demonstrated in languages that use duration as a cue to segmental contrasts, such as vowel length distinctions. German, for instance, distinguishes minimal word pairs differing in the vowel contrast /a/–/a:/ where words like *bannen*, “to banish,” contain a short /a/, and words like *bahnen*, “to channel,” contain a long /a:/ (without any major spectral differences; e.g., Reinisch, 2016a, 2016b). Critically, the perception of this vowel length contrast has been shown to depend on the speech rate of the preceding context. Listeners are more likely to interpret a vowel midway between /a/ and /a:/ as the long vowel /a:/ if it follows a context spoken at a fast rate, but as short /a/ if it follows a slow context (Reinisch, 2016a, 2016b). In other words, in rate-dependent perception a given duration is interpreted as contrasting with the preceding context. The effect of speech rate then is the difference in likelihood that a given sound (here: vowel) is perceived as long when following a fast versus a slow context. Rate-dependent perception has been shown in many different languages, affecting a wide range of temporal contrasts including vowel length distinctions in other languages (Gabay et al., 2019; Reinisch & Sjerps, 2013), voice onset time (VOT) of stop consonants (Kidd, 1989; Newman & Sawusch, 2009; Toscano & McMurray, 2015), formant transition duration (Wade & Holt, 2005), singleton versus geminates (Mitterer, 2018), and even the presence or absence of syllables or words (“lexical rate effect”—as compared with rate-dependent perception of phoneme contrasts; Bosker et al., 2020a; Brown et al., 2012; Dilley & Pitt, 2010; Kaufeld et al., 2020). Note that we name but a few recent examples of studies on rate-dependent perception and refer readers to Stilp (2020) for a comprehensive review.

Rate-dependent perception has been divided into effects of proximal, distal, and global context where proximal refers to the immediate context within approximately 250–300 ms around the target, distal refers to sentence-length context, and global to the experimental setting or general knowledge about a speaker (see, e.g., Maslowski et al., 2020, for definitions and discussion). The present study is concerned with sentence-length context, however, without distinguishing between proximal (immediately adjacent) and distal (longer, further removed) parts of the context sentences (for separate manipulations, see, e.g., Newman & Sawusch, 1996; Reinisch et al., 2011; Sawusch & Newman, 2000; Summerfield, 1981). Moreover, the present study is concerned with the rate-dependent perception of a durationally cued phoneme contrast (i.e., /a/–/a:/ in German; Reinisch, 2016a, 2016b) which some have argued to be qualitatively distinct from speech rate effects on lexical perception (i.e., dis/appearing function words in the lexical rate effect; Baese-Berk et al., 2019; Pitt et al., 2016).

Importantly, many experiments investigating rate-dependent perception used listening conditions that do not reflect what listeners typically experience in “real” life. Laboratory experiments tend to present an ideal (i.e., quiet) listening environment, to serve as a starting point to understand the workings of a given perceptual process. Still, an increasing body of literature is concerned with the need to understand speech perception in everyday communication involving possible listening adversities (for an overview, see, e.g., Mattys et al., 2012). Critically, it has been shown that speech perception does not always operate similarly in quiet compared with when listeners are confronted with challenging listening situations. Listeners flexibly adapt to different listening conditions and reweigh their reliance on different types of information accordingly (e.g., up- or down-weighing the use of acoustic, phonotactic, and lexical information; Derawi et al., 2022; Mattys, 2004; Mattys et al., 2009; Reese & Reinisch, 2022; Strauss et al., 2022; or the extent of considering alternative lexical candidates; Brouwer & Bradlow, 2016; McQueen & Huettig, 2012). Therefore, in order to explain the workings of speech perception in general and specific processes such as rate-dependent perception in particular, an assessment of its operation under different listening conditions is critical.

As for quiet listening conditions, the literature has shown that rate-dependent perception of phoneme contrasts is a low-level process that operates during early stages of speech perception. This is supported by findings that also non-speech contexts, such as pure tones or sine wave speech can trigger the effect (Bosker, 2017; Diehl & Walsh, 1989; Gordon, 1988; Wade & Holt, 2005), that the effect occurs very rapidly (Maslowski et al., 2020; Reinisch & Sjerps, 2013; Toscano & McMurray, 2015) and appears to operate prior to other early perceptual processes, such as stream segregation (Newman & Sawusch, 2009). In fact, speech rate information from competing speakers (e.g., in a cocktail party setting) cannot be ignored (Bosker et al., 2020a). This early use of rate information and its relative independence of the context being (intelligible) speech are some of the factors that have been claimed to differentiate rate-dependent perception of phoneme contrasts from the lexical rate effect (Bosker, 2017; Pitt et al., 2016). That is, the lexical rate effect tends to occur considerably later during processing (Brown et al., 2012; Brown et al., 2021; Maslowski et al., 2020) and critically depends on the context’s intelligibility (Pitt et al., 2016).

As for challenging listening conditions, rate-dependent perception has already been shown to be robust when listening to a speaker with a foreign accent (Bosker & Reinisch, 2015), when listening in a second language (Bosker & Reinisch, 2017), and even when simultaneously performing a secondary task (Bosker et al., 2017). That is, under all these conditions listeners continue to use the speech rate of a context sentence to interpret upcoming temporal cues to speech sounds, and importantly the speech rate effect is not reduced relative to the respective control conditions (i.e., native speech; low cognitive demands). However, how rate-dependent perception operates under conditions of energetic masking of the signal, for instance in noise, or other types of distortion, such as reverberant environments, remains unknown.

One repeated finding of studies on rate-dependent perception in adverse conditions was that when processing resources were taxed, either by listening in a second language (Bosker & Reinisch, 2017), or when performing a concurrent visual search task while listening to the context (Bosker et al., 2017), listeners responded to the target sounds as if the context was faster than without cognitive load. Since listeners typically also give higher speech rate estimates in explicit judgment tasks under cognitive load (Bosker & Reinisch, 2017), this was interpreted with regard to the mechanism how speech rate of the context is calculated. Specifically, two previous accounts of perceptual encoding in adverse listening conditions were tested. What we termed “noisy encoding” (Mattys & Wiget, 2011) due to general reduction in the robustness of processing of the speech signal, and “shrinking of time” (Casini et al., 2009; Chiu et al., 2019) due to impaired temporal sampling of the sensory input. Results suggested that with reduced cognitive resources being available for speech perception, listeners appear to miss temporal pulses, and thus underestimate durations. In other words, cognitive load makes speech sound fast (see Bosker et al., 2017, for a discussion). This finding together with the lack of reduction of the rate effect was interpreted as evidence for the “shrinking of time” account.

Mechanistically, the temporal sampling that underlies rate-dependent perception may involve entrainment of neural oscillations. The listening brain has been shown to “track” the syllabic rate of speech by phase-locking endogenous theta oscillations (i.e., 3–9 Hz) to the amplitude envelope of speech (Doelling et al., 2014; Peelle & Davis, 2012). These rate-dependent neural oscillations have been suggested to support speech intelligibility when “in sync” with the speech amplitude fluctuations (van Bree et al., 2021), in line with earlier demonstrations of the critical contribution of slow amplitude modulations in speech to intelligibility (Drullman et al., 2014a, 2014b; Fogerty & Humes, 2012). Specifically, ongoing oscillations are proposed to build temporal predictions about upcoming sensory input. In fact, experiments using magnetoencephalography (MEG) and transcranial alternating current stimulation (tACS) point towards a causal role of speech-tracking oscillations in the theta range in rate-dependent perception: participants who show greater evidence for neural entrainment to a context speech rate in MEG also demonstrate larger rate effects in behavior (Kösem et al., 2018). There are even indications that tACS can serve as external “pacemaker,” guiding the phase and frequency of endogenous oscillations, in turn influencing behavioral speech perception (Kösem et al., 2020; Riecke et al., 2018; Zoefel et al., 2018). In line with these neurobiological findings, behavioral rate-dependent effects are observed only for speech rates in the 3–9-Hz range—that is, when the speech rate can be encoded by ongoing theta oscillations (Bosker & Ghitza, 2018). Further behavioral support comes from the observation that special populations known to demonstrate neural entrainment impairments such as individuals with developmental dyslexia (Goswami, 2011; Goswami et al., 2002) also show a reduced rate effect relative to typically developed listeners (Gabay et al., 2019).

The neural tracking of speech is clearly susceptible to influences from the listening conditions: it is strongly reduced when listening in noise, in competing speech, and in real world acoustic scenes—relative to in quiet (Fuglsang et al., 2017; Rimmele et al., 2015; Zion Golumbic et al., 2012). This reduction in neural tracking reflects the behavioral listening challenges posed by, for instance, background noise and reverberant room acoustics (e.g., Fogerty et al., 2020; Helfer, 1994; Nábělek, 1988). Nevertheless, except for the most extreme circumstances, human speech comprehension typically does not break down entirely in challenging listening conditions. For instance, in noisy or multitalker situations, theta oscillations are often still successful at tracking the dynamics of attended speech (Ding & Simon, 2012; Mesgarani & Chang, 2012; Zion Golumbic et al., 2012). Similarly, the human brain is capable of compensating for reverberation, with speech envelopes reconstructed from EEG responses to reverberant speech resembling the original “clean” speech more than the reverberant stimulus (Fuglsang et al., 2017). This raises the question how robust the neural oscillatory mechanism that underlies rate-dependent perception is against noise and reverberation: Is rate-dependent perception modulated by challenging listening conditions?

Therefore, the present study investigates how listeners encode the temporal information of speech when the signal is degraded by noise or reverberation. This question is tested using a rate-dependent perception paradigm with a phoneme contrast as target: German listeners were presented with three sentences played at either a fast or slow speech rate, followed by target words sampled from an /a/–/a:/ vowel duration continuum (e.g., *bannen* vs. *bahnen*). We predicted that a fast context sentence should increase the probability of participants reporting *bahnen* with long /a:/, while the same target word should be more likely to be perceived as *bannen* with short /a/ if embedded in a slow speech rate (a typical rate effect). Critically, we applied two types of non-linguistic signal degradation: white noise mixed with the speech signal at 0 SNR and reverberation simulating a “big room” (see Methods for details). Note that we used relatively “moderate” degrees of signal degradation, challenging listening while maintaining intelligibility, as corroborated by ceiling performance on a separate intelligibility test (for details see the documents on OSF [https://osf.io/4fgkz/]). Consequently, we could in principle predict that the rate effect will not be affected by our two types of “moderate” signal degradation. This prediction would be supported by earlier claims that rate-dependent perception “is driven by a timing mechanism that requires hearing input as intelligible speech” (Pitt et al., 2016, p. 343). Note however that this claim contradicts evidence for rate effects induced by nonspeech, such as fast versus slow tones (Bosker, 2017). Still, we could speculate that as long as the signal degradation does not impact intelligibility, the rate effect should remain stable.

Alternatively, the signal degradations could have similar effects on rate-dependent perception as increased cognitive load. According to the “shrinkage of time” account, the same speech is perceived as faster under high versus low cognitive load, which may apply likewise to forms of *perceptual* load, such as signal degradation. This would predict an overall increase in long /a:/ responses in conditions of signal degradation compared with in quiet. This prediction is supported by the claim that “energetic masking not only critically impairs lexical access, it also decreases the size of the time window over which information is integrated” (Mattys et al., 2009, p. 233), hence speeding up the perceived tempo. Note that this prediction applies to stimuli in which *only the context is degraded* but not the target word (as in Bosker et al., 2017). In contrast, if signal degradation would be applied to the entire stimulus (context *and* target), the speeding up of the perceived tempo would presumably apply to both contexts and targets, removing the perceptual tempo difference between context and target.

Finally, the signal degradation could also induce “noisy,” less precise temporal encoding of the speech rate, triggering a reduction of the rate effect in degraded speech versus quiet. Note that the two types of signal degradation—noise and reverberation—were chosen to compare their specific characteristics with regard to the way they distort the signal. White noise with its uniform spectrum and a lack of amplitude modulation was taken as a baseline for overall energetic masking of the signal. Since humans do not perceive all frequencies equally, white noise applies masking of all frequencies while not interfering with these natural perceptual nonlinearities. Its masking of the spectral information should reduce the overall clarity of the speech signal. Poorer access to spectral information might consequently disrupt the encoding of temporal information needed to calculate speech rate and in this way lead to reduced rate effects on the categorization of a target word. Reverberation, in contrast, involves reflections of sound from the room’s walls and surfaces that mix with the direct sound source, specifically inducing changes in the signal’s temporal envelope (Houtgast & Steeneken, 1973). This could more directly impair the encoding of the temporal dimension of speech, possibly in the form of reduced entrainment of neural oscillations, and hence reduce the rate effect.

However, listeners have also been shown to rapidly adapt to signal degradation, learning to overcome the listening challenge offered by persistent noise or reverberation after some exposure. This is evidenced, for instance, by intelligibility improvements over the course of speech-in-noise exposure, asymptoting after as few as 15 sentences; Cainer et al., 2008). This is also in line with neurobiological evidence that not only nonprimary but also primary auditory cortex show invariance to stable background noise (Kell & McDermott, 2019; Mesgarani et al., 2014). Human neural responses to abrupt changes in background noise show rapid and selective suppression of the acoustic characteristics of the speech-masking noise in as little as 1 second after noise onset (Khalighinejad et al., 2019). Considering this rapid adaptation to background noise, perhaps listeners are capable of quickly compensating for the masking noise in the present rate-dependent perception experiments, much like how humans learn to adjust their rate perception to atypical noise-vocoded input (Jaekel et al., 2017; Shannon et al., 1995), hence predicting similar rate effects in noise compared with in quiet.

Similarly, listeners can also adapt to reverberant environments (Beeston et al., 2014; Srinivasan & Zahorik, 2013; Stilp et al., 2016; Watkins, 2005; Watkins et al., 2011; Watkins & Makin, 2007). For instance, Watkins (2005) tested the perception of an English *sir–stir* continuum, which is mainly cued by the closure duration of the /t/ in *stir* (i.e., longer closure suggests the presence of a /t/). He showed that adding reverberation to the target word continuum shifts the categorization boundary towards more *sir* responses. This suggests that listeners perceptually incorporate the reverberation with the sound such that the added “tail” from reverberation is fused with the actual sound obscuring the (closure of) /t/. Critically, this effect was reduced if the target word was embedded in a reverberant sentence context suggesting that information from the context could be used to compensate for the masking effect on the target. This compensation for context even held across “changes in room” (i.e., specific characteristics of the reverberation; Watkins, 2005) and has been shown to depend on the temporal envelope rather than temporal fine structure of the context (Watkins et al., 2011). For the present question about rate-dependent perception when confronted with distorted speech, this ability to compensate for the consequences of reverberation and specifically its connection to the temporal envelope might interact with the predicted reduction of the rate effect due to distortion of the context. Therefore, how reverberation and background noise affect rate-dependent perception remains an intriguing question that lies at the intersection of listener normalization for prosodic variability (here: speech rate) and listener adaptation to challenging listening conditions.

In sum, in the present study we investigated the effect of rate-dependent perception in degraded listening conditions, specifically under two types of signal degradation, white noise and reverberation, compared with a “clear” condition forming the baseline without signal degradation. In order to compare the impact of different types of contexts on the same target stimuli, in Experiment 1 only the context sentences but not the target words were subjected to signal degradation. This design matches previous studies on rate-dependent perception where responses to identical targets were compared across conditions (i.e., most studies on the phonemic rate effect discussed above, e.g., Bosker et al., 2017; Reinisch, 2016a, 2016b). Since, however, in the present study such an abrupt change from noisy or reverberant context to a clear target may seem unnatural, Experiment 2 compared rate effects across conditions where contexts and targets were coherent in terms of signal quality. This allows for detecting potential effects of adaptation to degraded listening conditions on rate-dependent perception.

## Experiment 1

### Method

#### Participants

Participants were recruited via the web-platform^1^ Prolific (www.prolific.co) [in February 2021] and were paid for their participation. In order to be eligible for the study, they were required to be a native speaker of German living in Germany, be between 18 and 50 years of age, use a desktop computer rather than their cellphone or a tablet, and wear headphones. Based on the number of participants in comparable previous studies (e.g., Bosker et al., 2017), 50 participants were recruited (27 female, 23 male), though data from one participant were excluded from analyses since this person reported in a postexperiment questionnaire to have stopped doing the task properly at some point during the experiment. Participants’ mean age was 28.5 years (*SD* = 7.5). They all confirmed to meet the criteria of being native speakers of German, and to have no history of hearing impairment or dyslexia. Nineteen reported to use over-ear headphones, nine on-ear, and 22 in-ear headphones. All participants gave informed consent to participate. The study was carried out in accordance with the research guidelines of the funding organization (German Research Council) and the requirements for good practice of the online platform (www.prolific.co) that was used for recruitment.

#### Materials

Stimuli were taken from a previous study (Reinisch, 2016b). Three German minimal word pairs differing minimally in the /a/–/a:/ vowel duration contrasts were selected as targets (*bannen–bahnen*, “banish”–“to channel”; *rammen–Rahmen*, “drive by impact”–“frame”; *Ratte–Rate*, “rat”– “installment”). Each target pair had been recorded in a different carrier sentence that did not contain any tokens of the two critical vowels. Those unique context-target pairings were kept for the present experiments. However, targets and sentences were manipulated separately before being spliced back together. In addition to using three context-target pairings, materials from two speakers were used. Both speakers were young female adults and native speakers of Standard German. Both voices had already been used in the previous study (Reinisch, 2016b), where the procedure of stimulus selection, manipulation of the duration continuum and speech rate manipulation of the context, as well as pretests are reported in detail.

In short, the /a/–/a:/ vowel duration continua were created by starting with the two speakers’ average duration of the long vowel for each word pair and subsequently creating 16 shorter continuum steps by using the duration tier in PRAAT (Boersma & Weenink, 2009) and PSOLA (pitch-synchronous overlap-add) resynthesis. The short endpoints were at the average duration of the speakers’ short vowels. All other segments in the words were set to an average value between the two speakers’ segments averaged over the words with the long and short vowel. The sentences were also manipulated using PSOLA to create two different rate conditions. For the fast rate condition, the entire sentences (though without targets) were compressed on an individual basis to be 15% faster than original recordings (resulting approximately in a rate of six syllables per second); and for the slow condition, sentences were expanded to be 10% slower than original (approximately 4.6 syllables per second). Two pretests then determined which part of the vowel duration continuum in the targets was suitable to yield responses from clearly more “long vowel” responses to clearly more “short vowel” responses without including steps where listeners would perform at ceiling. Based on the pretests reported in Reinisch (2016b), five continuum steps were selected per word pair for the present study. These were also the five middle steps used in the previous study and ranged from 107 to 149 ms for *bannen-bahnen* and *rammen–Rahmen*, and from 95 to 129 ms for *Ratte–Rate*. Note that different values and ranges result from differences in the phonological context in the words (i.e., vowel followed by a nasal vs. stop) and how natural a given manipulation sounded. These values were identical for the two speakers. The pretests also determined that the rate manipulation of the context sentences was sufficiently strong to shift the perception of the vowel duration depending on the context rate. For the minimal word pairs and continuum steps selected for the present study, the difference in “long vowel” responses following the fast versus slow contexts was 15%.

**Fig. 1:**
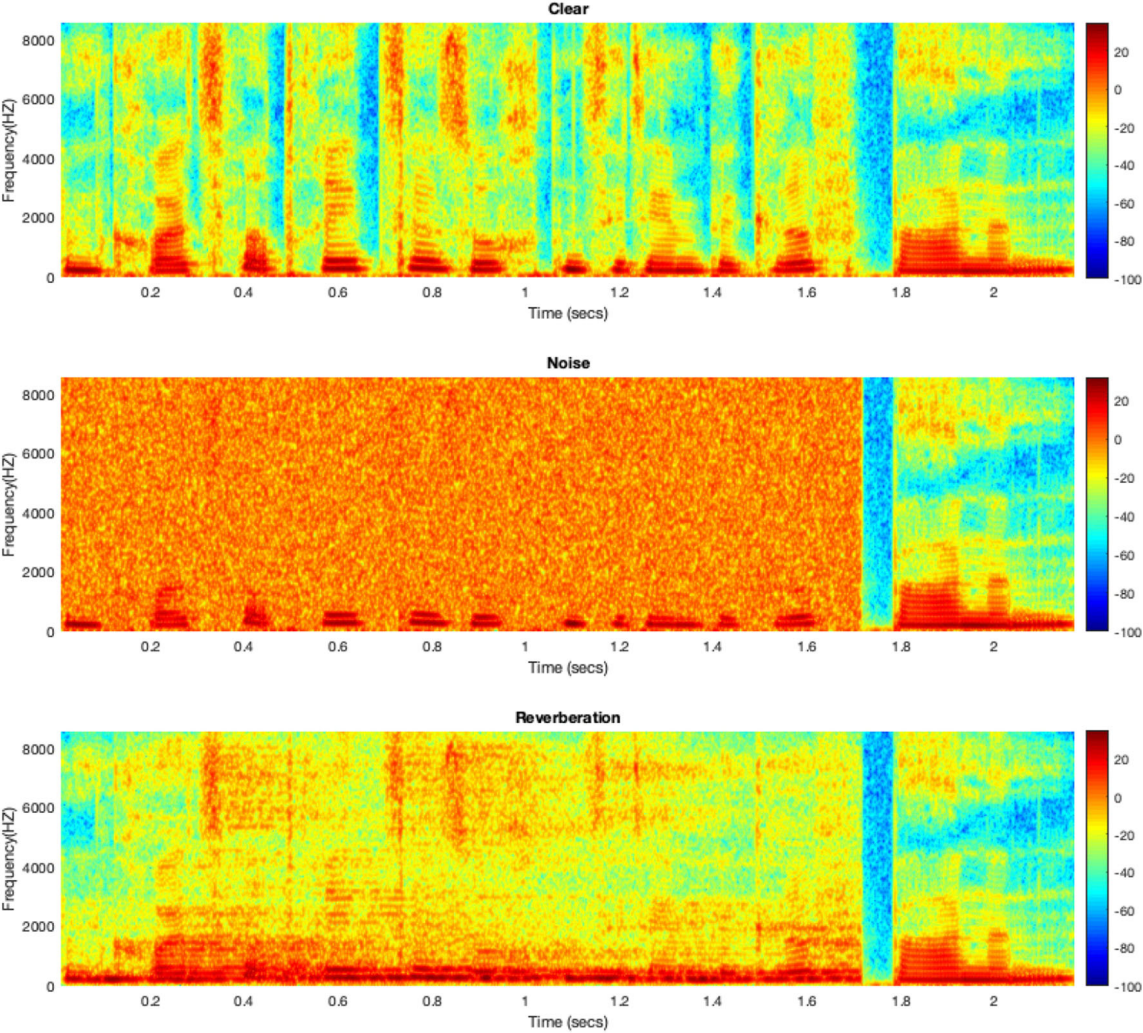
Broadband-spectrograms of the three conditions for Speaker 0 and the context sentence “Im Kreuzworträtsel suchten sie den Begriff ...” (In the crossword puzzle they were looking for the term ...) at the fast rate. The target is the middle step of the “bahnen/bannen” vowel duration continuum. Target onset is at 1.7 s and is indicated by the vertical band showing low energy which amounts to the closure of the /b/. The top panel shows the clear context, the middle panel the noise context, and the bottom panel the context with reverberation. The *x*-axis shows the time from sentence onset in seconds, the *y*-axis the frequency range from 0 to 8000 Hz and shading shows energy at a given point in time at a given frequency band (color online). Note that in Experiment 1 only the context sentences were degraded while the target was “clear” (as shown here), while in Experiment 2 the signal degradation was applied to the entire stimulus

For the present study, these baseline stimuli formed the “clear” condition. This clear condition was further manipulated to create the noise and reverberation conditions. First, the complete sentences including the targets at different vowel duration steps were manipulated. Note that this resulted in degraded context sentences including the targets. However, the goal of Experiment 1 was to test the effect of signal degradation on the context sentences only. Therefore, the manipulated targets were spliced off and replaced by the targets from the baseline condition (i.e., no manipulation). No silent interval was left between carrier sentence and target. Figure 1 shows the spectrograms of the three conditions in Experiment 1.

For the noise manipulation, an existing PRAAT script^2^ was used and further adapted by the first author such that it mixed all speech sound files with the same predefined sound file containing white noise at an SNR of 0. White noise was chosen to physically mask all frequencies equally while leaving the natural differences of perceiving different frequencies intact. The SNR was chosen such that the noise was clearly audible and potentially interfering but the sentences were still intelligible. For the reverb manipulation the vocal toolkit plugin (Corretge, 2012–2021) in PRAAT was called via a script written by the first author. The plugin allows to add reverberation to each sound file by convolution with an impulse response file that is provided by the toolkit. The option “Room Big”^3^ was selected at a mix of 50%. This resulted in well-audible reverberation while keeping a reasonable level of intelligibility. Finally, all stimuli were normalized for RMS amplitude. Example stimuli for all conditions can be found on the Open Science Forum (OSF; https://osf.io/4fgkz/). Note that the levels of noise and reverberation were chosen specifically to challenge listening while maintaining intelligibility. A separate intelligibility test with new German-speaking participants confirmed ceiling performance with these signal distortions (i.e., 99% correct in quiet, 98% in noise and 99% in reverberation; see OSF [https://osf.io/4fgkz/] for details). Thus, the present study serves as a starting point for exploring potential effects of signal degradation on rate-dependent perception.

#### Design and procedure

Stimuli were presented blocked by context condition with order of blocks roughly counterbalanced across participants. In the end, 7-9 participants completed each of the six possible orders. The slight imbalance was caused by the automatic assignment of block orders that did not account for participants who did not complete the experiment and were hence not included in the present dataset. Within each block, all stimuli were presented in fully random order (speakers, sentences/targets, rates, continuum steps) twice with the restriction that all stimuli had to be presented once before being repeated. The experiment was implemented in the Gorilla Experiment Builder (www.gorilla.sc), an online platform supporting web experiments.

Participants were instructed by means of written text that on each trial they would be presented auditorily with a sentence ending in a word that might sound ambiguous between two options. Their task was to indicate by button-press which of the two possible options they heard. For each sentence, the two possible target words (of the minimal pair) were presented visually on the screen with the letters “f” and “j” written underneath the words. These were the buttons that participants were asked to press on their computer keyboard to indicate their choice. They should press “f” if they thought they heard the word on the left, and “j” if they thought they heard the word on the right. The word with the long vowel was always presented on the right, so any potential bias was the same across conditions and experiments. On each trial, the text appeared at the same time as the audio started playing and stayed on the screen until the response was logged by button press. The next trial started automatically after 1,000 ms.

Participants were informed up front that some of the stimuli might sound “noisy” but they should ignore this noise. They received three practice trials, randomly sampled from the main experiment but identical for all participants, one in each condition, in the order clear context, noise, reverberation. After these practice trials, participants were asked to adjust the sound level of their computer to a comfortable level such that they won’t need to change it anymore during the experiment. After another three (randomly selected) practice trials, they were informed that now they were not supposed to change the volume anymore for the rest of the experiment. The experiment started by pressing space bar. Between blocks as well as once within each block, participants were allowed to take a self-paced break. The experiment consisted of a total of 360 trials and took approximately 30 minutes to complete.

### Results

Statistical analyses were conducted using linear mixed-effects models as implemented in the lme4 package (Bates et al., 2015) in R (Version 4.0.3; R Core Team, 2020) using a logistic linking function (Jaeger, 2008) to account for the binomial nature of our dependent variable, which was Response, with the long vowel /a:/ coded as 1 and the short vowel /a/ coded as 0. Fixed effects were Continuum Step, Speech Rate, Condition, and all interactions. In addition, Speaker was modeled as a covariate since an exploratory model-fitting procedure using log-likelihood ratio tests suggested a significant improvement of model fit when Speaker (contrast coded to −0.5 and 0.5) was included. Note that the inclusion of the covariate does not affect the interpretation of our main factors of interest (i.e., Continuum Step, Speech Rate, Condition) since those are modeled with regard to the mean of the levels of the covariate. The additional inclusion of trial number within each block (centered and rescaled) as a covariate did not improve the model fit. The same held for the inclusion of block order (six levels) which additionally led to convergence issues. Hence, neither Trial Number nor Block order were included as covariates in the final model.

Of the fixed factors of interest, Continuum was entered as a continuous variable coded to be centered on zero (i.e., subtracting the mean), Speech Rate was contrast coded to fast rate coded as 0.5, and slow rate coded as −0.5. Condition was factor coded with the level clear context mapped onto the intercept (as it serves as a baseline), and contrasts being reported for clear versus noise, and clear versus reverberation.

The random-effects structure included a random intercept for participants. Random slopes were then added one at a time and kept in the model if they significantly improved the model fit as determined by model comparisons using log-likelihood ratio tests. We report the best fitting model that converged and did not give us a singularity error. Unless noted otherwise, random slopes for Continuum Step, Speaker, Condition, and Speech Rate were included. Note that a random intercept over items was not included, since any single factor contributing to variability in items had too few levels as to be meaningful as a random factor.

**Fig. 2:**
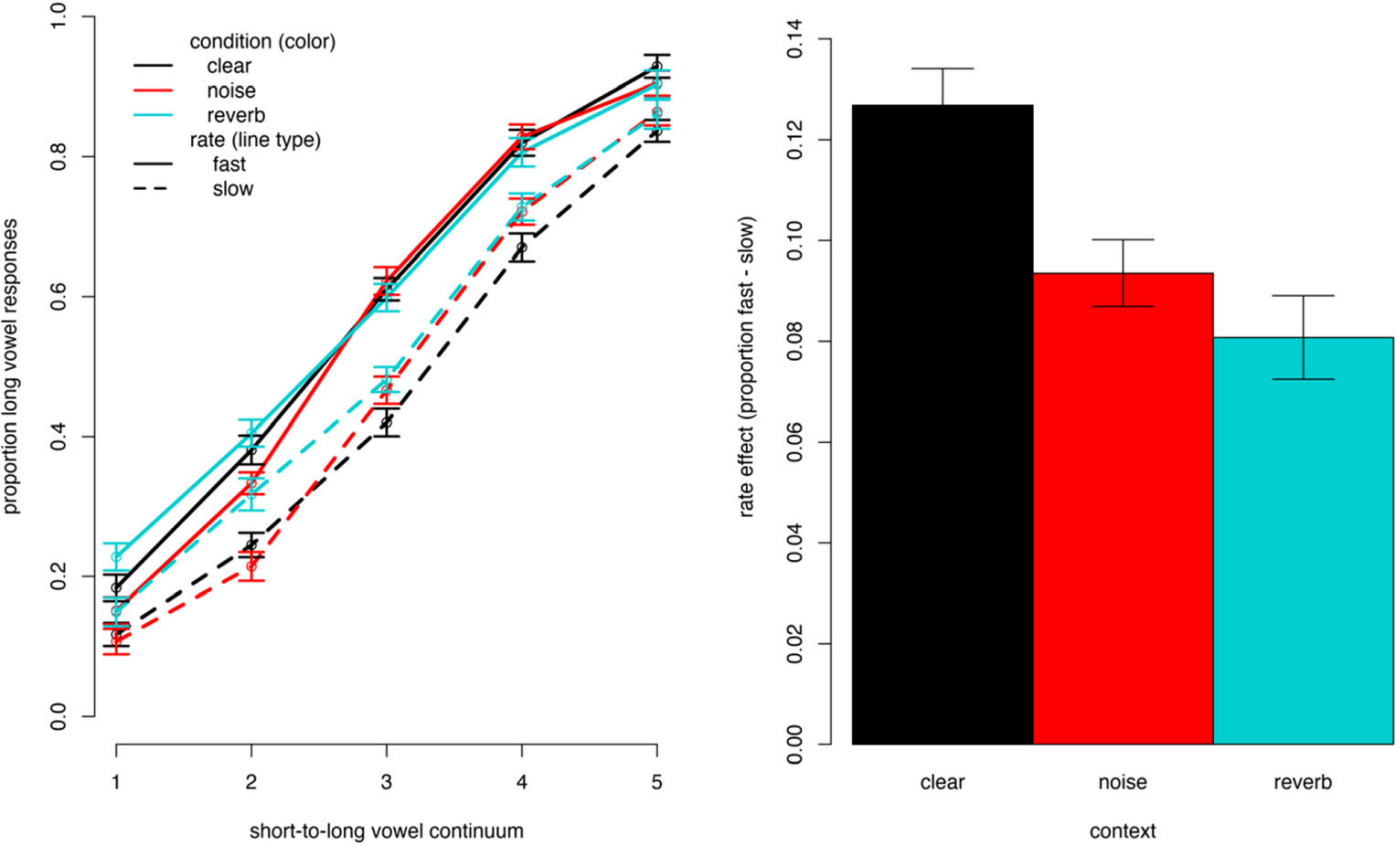
Visualization of results of Experiment 1. The left panel shows the categorization functions (lines) based on the raw data (dots) representing the proportion of long-vowel responses over continuum steps (the higher the step the longer the vowel). Responses following a fast context are represented as solid lines; responses following a slow context are shown in dashed lines. The colors (online) represent the conditions with black = clear, red = noise, turquoise = reverberation. The right panel shows the rate effect in the different context condition as measured by the difference in long-vowel responses following fast versus slow contexts. Color coding (online only) is the same as for the left panel. The error bars in both panels show one standard error, taking into account the within-participant design of context condition

The results of the final model are listed in Table 1, and the rate effects across conditions are illustrated in Fig. 2. The factors that are mapped onto the intercept hold for the clear Condition and show effects of Speech Rate with more long-vowel responses following a fast versus slow context (typical rate effect), an effect of Continuum with more long-vowel responses for longer vowels. Critically, a number of interactions was found. Specifically, the interaction between ConditionNoise and Rate as well as the interaction between ConditionReverb and Rate demonstrated that the rate effect was smaller in the two degraded speech conditions as indicated by the negative estimate. Additionally, the effect of Continuum differed between the clear and the reverberation context such that the effect of Continuum was smaller, that is, the categorization slope was shallower, following the reverberation than the clear context.

**Table 1:** Results of the fixed effects of the statistical model for Experiment 1

	*b*	*SE*	*z*	*p*
Intercept	0.15	0.10	1.40	.161
ConditionNoise	0.00	0.08	0.09	.931
ConditionReverb	0.17	0.09	1.82	.069
Rate	0.91	0.09	10.63	.000
Continuum	1.17	0.06	18.80	.000
Speaker	−0.65	0.14	−4.67	.000
ConditionNoise:Rate	−0.21	0.10	−2.15	.031
ConditionReverb:Rate	−0.34	0.10	−3.60	.000
ConditionNoise:Continuum	0.07	0.04	1.80	.071
ConditionReverb:Continuum	−0.11	0.04	−2.74	.006
Rate:Continuum	0.10	0.06	1.74	.08
ConditionNoise:Rate:Continuum	−0.07	0.08	−0.90	.368
ConditionReverb:Rate:Continuum	−0.10	0.08	−1.25	.210

*Note.* The clear context condition was mapped onto the intercept and other effects are to be interpreted relative to this reference level

Additional analyses using statistically equivalent models with the factor Condition coded such that the levels noise and reverberation were mapped onto the intercept showed that despite the reduction of the rate effect relative to the clear condition found in the main model, effects of rate were found for each of these conditions—reference is noise: *b*_(Rate)_ = 0.70, *SE* = 0.09, *z* = 8.17, *p* < .001; reference is reverberation: *b*_(Rate)_ = 0.57, *SE* = 0.08, *z* = 6.80, *p* < .001. Furthermore, even though we did not set out to match and compare the two degraded context conditions directly, the additional models suggest that the magnitude of the decrease in the effect of rate did not differ between the noise and reverberation condition relative to the clear condition—reference is noise: *b*_(Rate:conditionReverb)_ = −0.13, *SE* = 0.10, *z* = −1.40, *p* = 0.162. This opens the issue for future studies to address in more detail how rate-dependent perception changes not only in different types but under different degrees of signal degradation. The dataset, code, and results for all models can be found on OSF (https://osf.io/4fgkz/).

### Discussion

Experiment 1 tested the effect of signal degradation on rate-dependent perception of a German vowel duration contrast. We found that relative to a clear context (without degradation) the rate effect as shown by the difference in proportion of long-vowel responses in a time-compressed fast versus time-expanded slow context sentence was smaller when the context sentence was masked by white noise or degraded by reverberation. Note that both types of signal degradation were applied *to the context sentences only*; the target words were always presented without degradation (see Fig. 1). Hence, the reduced rate effect in noisy and reverberant contexts suggests that the signal degradation hindered the uptake of information relevant to the calculation of speech rate. The implications with regard to accounts of speech perception in degraded listening conditions will be discussed in the General Discussion.

With regard to the perception of the vowel duration continuum, results showed differences between the clear and the reverberation context condition. A flatter categorization curve of the continuum was found following the context with reverberation. Since reverberation tends to smear spectral information over time, it likely reduces the possibility for the extraction of precise temporal cues. This could have impacted the reliance on the actual vowel duration during target categorization, lowering perceptual precision.

However, the main goal of Experiment 1 was to assess the magnitude of the rate effect on identical targets following different types of contexts. To achieve this goal, we varied the signal degradation in the context while keeping the target words constant (i.e., always clear). As a result, the coherence of the signal between context and target differed across conditions. While one could imagine a loud noise to stop abruptly while listening to speech or one moving outside a reverberant environment, it is evident that the clear context condition was the most natural one with regard to coherence between context and target. This raises the question how the outcomes of Experiment 1 generalize to more naturalistic listening conditions, where signal degradations are typically relatively stable.

In order to address this issue of acoustic coherence between context and target, Experiment 2 was designed to test rate-dependent perception when not only the context sentences but also the targets were degraded by noise or reverberation. As has already been discussed in the introduction, listeners have been shown to compensate in perception for degraded listening conditions involving noise (Cainer et al., 2008; Kell & McDermott, 2019; Khalighinejad et al., 2019; Mesgarani et al., 2014) or reverberation (Beeston et al., 2014; Srinivasan & Zahorik, 2013; Stilp et al., 2016; Watkins, 2005; Watkins et al., 2011; Watkins & Makin, 2007). If acoustic coherence between context and target allows for compensation for the degradation then the rate effect may not be reduced relative to a clear context in Experiment 2.

## Experiment 2

### Method

#### Participants

Participants were recruited via the web-platform Prolific (www.prolific.co) [in February 2021] according to the same criteria as those for Experiment 1 but with the requirement to not have participated in the other experiment. They were paid for their participation. Again, informed consent was obtained and the experiment was carried out in accordance with the research guidelines of the funding organization (German Research Council) and the requirements for good practice of the online platform (www.prolific.co) that was used to recruit participants. Forty-eight participants (21 female, 26 male, one did not say) took part, roughly matching the overall sample size of Experiment 1. Participants’ mean age was 30.4 years (*SD* = 6.9). In a post-experiment questionnaire, they all confirmed to be native speakers of German, and to have no history of hearing impairment or dyslexia. Twenty-one reported to use over-ear headphones, 10 on-ear, and 17 in-ear headphones.

#### Material, design, and procedure

Materials were close to identical to Experiment 1 with the only difference that after the addition of noise or reverberation for the degraded context conditions, targets were not spliced off and hence not replaced by the targets from the clear condition. Instead, the fully manipulated sentences were kept such that the manipulation of condition was coherent between context and target. Design and procedure of the experiment were identical to Experiment 1. Context conditions were blocked with possible orders roughly counterbalanced across participants, again resulting in 7–9 participants per block order, according to the same sampling algorithm as discussed above.

### Results

**Fig. 3:**
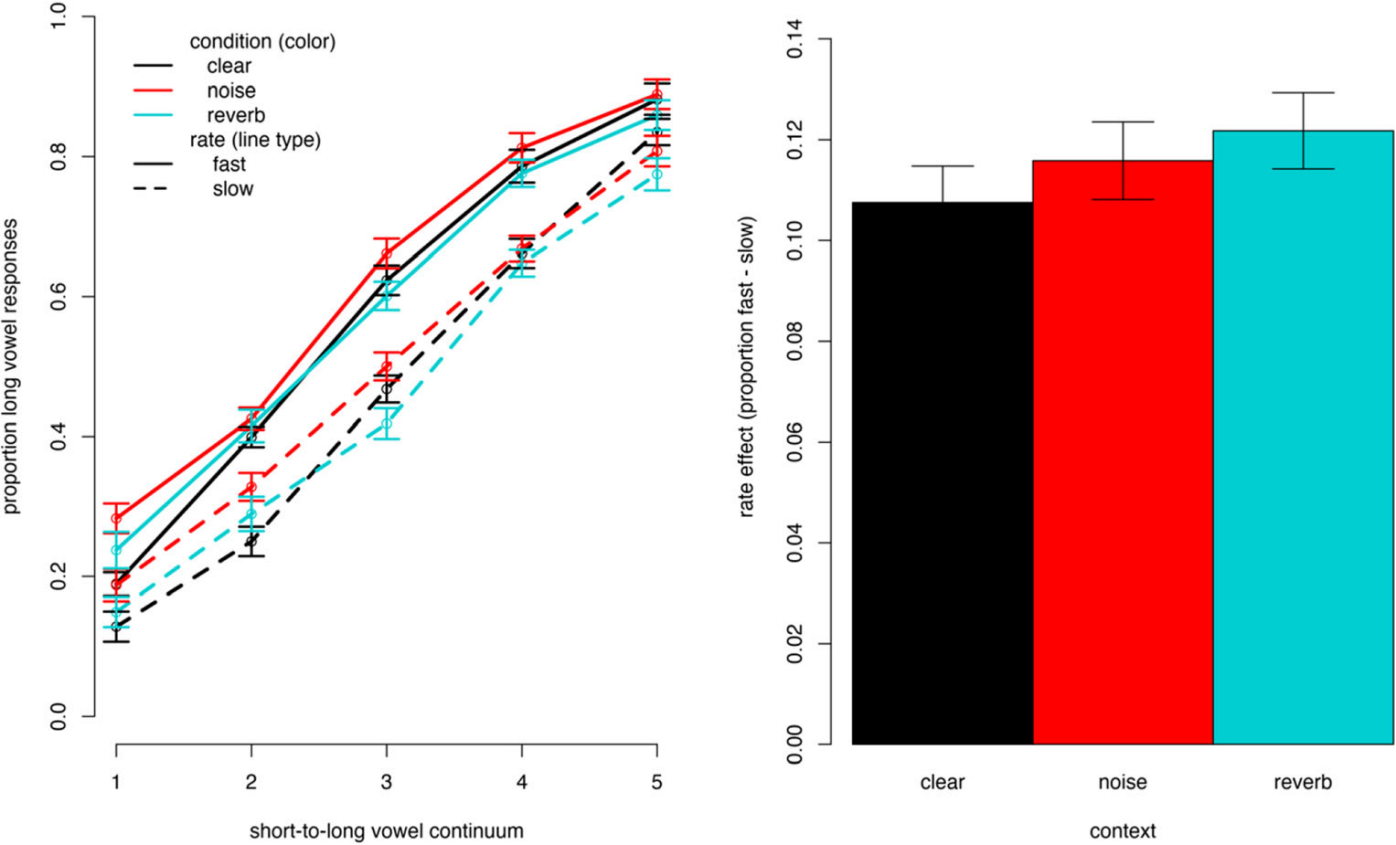
Visualization of results of Experiment 2. The left panel shows the categorization functions (lines) based on the raw data (dots) representing the proportion of long-vowel responses over continuum steps (the higher the step the longer the vowel). Responses following a fast context are represented as solid lines; responses following a slow context are shown in dashed lines. The colors (online) represent the conditions with black = clear, red = noise, turquoise = reverberation. The right panel shows the rate effect in the different context condition as measured by the difference in long-vowel responses following fast vs. slow contexts. Color-coding (online only) is the same as for the left panel. The error bars in both panels show one standard error, taking into account the within-participant design of context condition

Data were analyzed using the same generalized-linear mixed-effects model as described Experiment 1 except for the random slope for Speech Rate over participants that had to be dropped because it gave us a singularity error. Data and analyses are available on OSF (https://osf.io/4fgkz/). The covariates trial number within each block and block order also did not improve the model fit for Experiment 2 and were hence not included. Results are shown in Table 2 and rate effects across conditions are illustrated in Fig. 3. As in Experiment 1, for the clear Condition that was mapped onto the intercept, significant effects were found for Speech Rate (more long-vowel responses following a fast than slow rate) and Continuum (more long-vowel responses the longer the vowel). The effect of ConditionNoise suggests that more long-vowel responses were given overall in the noise than the clear condition. No such difference was found between the reverberation and clear condition. Importantly, as in Experiment 1, interactions indicate that effects found for the clear condition differed in the other two conditions. This was the case for the effect of continuum, where the negative regression weights for ConditionNoise:Continuum and ConditionReverb:Continuum suggest that the effect of continuum was smaller; that is categorization curves were shallower for these two conditions relative to the clear condition. Critically, however, in contrast to Experiment 1, the effect of Speech Rate in Experiment 2 did not differ between the clear condition and the two other context conditions. In a direct comparison,^4^ running an omnibus model on the data from both Experiment 1 and 2 (with Experiment 2 mapped onto the intercept), we observed a three-way interaction between ConditionReverb:Rate:Experiment 1. It suggests that the difference in the rate effect between the clear and reverberation context conditions was larger in Experiment 1 than Experiment 2, likely explaining the null result for the Rate:Condition interaction in Experiment 2.

**Table 2:** Results of the fixed effects of the statistical model for Experiment 2

	*b*	*SE*	*z*	*p*
Intercept	0.17	0.13	1.32	.187
ConditionNoise	0.20	0.08	2.70	.007
ConditionReverb	−0.06	0.12	−0.49	.625
Rate	0.71	0.07	10.36	.000
Continuum	1.13	0.07	16.86	.000
Speaker	−0.86	0.12	−6.81	.000
ConditionNoise:Rate	0.01	0.10	0.16	.877
ConditionReverb:Rate	0.06	0.10	0.67	.505
ConditionNoise:Continuum	−0.17	0.04	−4.33	.000
ConditionReverb:Continuum	−0.14	0.04	−3.49	.000
Rate:Continuum	−0.01	0.05	−0.20	.842
ConditionNoise:Rate:Continuum	0.09	0.07	1.23	.220
ConditionReverb:Rate:Continuum	0.02	0.07	0.32	.750

*Note*. The clear context condition was mapped onto the intercept and other effects are to be interpreted relative to this reference level

### Discussion

Experiment 2 tested rate-dependent perception of speech in white noise and with reverberation, in a situation where not only the context that provides the critical information about speech rate is degraded (as in Experiment 1) but also the to-be-recognized targets. While, unlike in Experiment 1, now the perception of physically different targets is compared, within each condition the context and target were coherent. We hypothesized that this continuity in signal (with regard to degradation) might allow for better grouping of context and target, and thereby, allowing for compensation mechanisms to operate. Previous studies on speech perception and comprehension in noise and reverberation have already shown that listeners use prior exposure to compensate for signal degradation (e.g., Cainer et al., 2008; Watkins, 2005). Results of Experiment 2 suggest that when context and target were coherent, the rate effect did not differ (i.e., decrease) under noise and reverberation relative to the clear sentence.

However, two differences are worthwhile mentioning here. Firstly, we found a main effect of ConditionNoise, with more long vowel responses in the noise than clear condition. While this is in line with the hypothesis that suboptimal listening conditions “make speech sound fast” (Bosker et al., 2017; Bosker & Reinisch, 2017), no such effect was found for the reverberation condition. Moreover, since no such difference in overall long-vowel responses between conditions was found in Experiment 1, we refrain from strong conclusions about this effect.

Interestingly, however, in both degraded context conditions the effect of continuum was smaller than in the clear condition; that is, identification functions were shallower. This finding is likely explained by the fact that when the targets are degraded, that is, either masked by white noise or distorted through reverberation, the vowel duration is also less easily perceived.

## General discussion

The present study tested rate-dependent speech perception under degraded listening conditions, specifically, when white noise or reverberation was added to the signal relative to a condition in which context and targets were presented in the clear. Previous research has shown that rate-dependent perception is a robust, low-level perceptual mechanism through which listeners take into account temporal properties (i.e., speech rate) of a context to interpret duration cues for spoken-word recognition (Bosker, 2017; Reinisch & Sjerps, 2013; Sjerps & Reinisch, 2015; Toscano & McMurray, 2015). However, previous studies have also suggested that listeners modulate their reliance on different types of information when confronted with adverse listening conditions (e.g., Derawi et al., 2022; Mattys et al., 2009; Strauss et al., 2022) suggesting that rate-dependent perception might be modulated if the signal is degraded.

Different possible accounts of rate-dependent perception under signal degradation, specifically in white noise and reverberation, were proposed. Firstly, if the effect of signal degradation was similar to the effect of taxing cognitive resources through a secondary task (i.e., since perception becomes harder overall) then the degraded signals could have led to the impression of the stimuli being overall faster. This would predict more long-vowel responses in the degraded conditions than in the clear. Such a finding has previously been interpreted as supporting a “shrinkage of time” account where reduced cognitive resources lead listeners to miss speech samples in the calculation of rate (Bosker et al., 2017). Alternatively, signal degradation could have led to what has previously been termed “noisy encoding,” since it literally obscures acoustic information that is required to calculate speech rate. This account predicts a reduction in the rate effect under conditions of signal degradation. Although these accounts are not mutually exclusive (see the discussion in Bosker et al., 2017), the present findings mainly support the “noisy encoding” account: a reduced rate effect in categorizing a (clear) vowel duration continuum when the context sentences are distorted by white noise or reverberation.

Experiment 1 compared rate-dependent perception across conditions when only the context was degraded, allowing for a comparison of the perception of acoustically identical targets. We found in both the white noise and reverberation condition that the rate effect was reduced relative to the clear-context condition, supporting the hypothesis of a reduced rate effect. As discussed in the introduction, the two types of signal degradation were chosen specifically to assess spectral distortion by the flat spectrum of white noise on the one hand, and the effect of temporal distortion through reverberation on the other hand. Both types of degradation were kept at a moderate level so as to avoid compromising the intelligibility of the sentences (which was confirmed in a separate intelligiblity test). Listeners were hence likely still able to access information about speech rate, for instance, by means of entrainment of neural oscillations. This presumably accounts for why we did still observe rate effects in degraded signal conditions, albeit in a reduced form. However, compared with situations where listeners were asked to divide cognitive resources during speech processing (Bosker et al., 2017) or experiments in which rate information was provided by non-speech context in the form of tones, here the signal that provided the rate information was less readily accessible though degradation.

Although differences between the two conditions of signal degradation could be predicted based on their specific characteristics of degrading the speech signal, the relative degree of (perceived) degradation between two conditions is hard to quantify. That is, in each condition, the signal is degraded in qualitatively distinct manners and—with ceiling intelligibility performance—it cannot be claimed that the two conditions equally affect perceptual processing. This is why we refrain from interpreting a direct comparison between these two context conditions, albeit, if one insists on such a comparison, the magnitude of reduction of the effect of rate appeared not to differ between the noise and reverberation context relative to the clear context in Experiment 1. Future studies may focus on a more thorough exploration of the effects of level of noise or reverberation, asking about thresholds of degradation when generally robust low-level processes such as rate-dependent perception start to lose impact until they completely diminish (cf. Bosker & Ghitza, 2018). The main finding of Experiment 1 of the present study was that signal degradation of a context can lead to a reduction of rate-dependent speech perception and might hence be qualitatively different from listening under taxed cognitive load with a clear speech signal (Bosker et al., 2017). Notably, this finding is in line with previous comparisons of effects of energetic masking (i.e., physical signal degradation) and informational masking involving the reduction of cognitive resources (Mattys et al., 2009).

Experiment 1 compared the impact of degraded context sentences on clear targets without signal degradation in order to compare responses to identical target stimuli. Experiment 2 then compared rate-dependent perception in different conditions where not only the contexts but also the targets were manipulated. While this necessarily means that we had to compare responses to acoustically different targets, the coherence between context and target was the same across conditions. Previous studies have shown that with coherent signals listeners are able to account for noise (Cainer et al., 2008; Kell & McDermott, 2019; Khalighinejad et al., 2019; Mesgarani et al., 2014) and reverberation (Beeston et al., 2014; Srinivasan & Zahorik, 2013; Stilp et al., 2016; Watkins, 2005; Watkins et al., 2011; Watkins & Makin, 2007) for speech comprehension and phonetic categorization. Based on these previous studies, discussed in the introduction, we hypothesized that the coherence between context and targets might allow listeners to compensate in perception for the signal degradation. Indeed, Experiment 2 with context and targets manipulated did not find a reduced rate effect in the two degraded conditions relative to the clear. Although this null effect for an interaction between Condition and Rate has to be interpreted with caution, it does provide some indication for the robustness of rate-dependent perception.

Note that the reduced rate effects in Experiment 1 (with incoherence between contexts and targets in signal degradation) but no reduction of the rate effect in Experiment 2 (with coherence in signal degradation) may be interpreted in two different ways. One could argue that rate-dependent perception operates most efficiently if the context and target can be perceptually grouped together (i.e., coherence). However, there is evidence against this premise in earlier literature. For instance, using different talkers in contexts versus targets does not modulate rate-dependent perception (Bosker, 2017; Maslowski et al., 2018). In fact, even the speech rate of an *unattended talker* in multitalker listening conditions has been found to influence the perception of targets produced by an attended talker (Bosker et al., 2020a). Therefore, we interpret the different outcomes of Experiment 1 and 2 in terms of noisy—that is, imprecise encoding of the temporal characteristics of the context. While Experiment 2 allowed for listener adaptation to the coherent signal degradations in contexts and targets, this was not the case in Experiment 1. As a result, the temporal properties of the context and target were more difficult to contrast for the listener, reducing the rate effects in Experiment 1. This is in line with findings that rate-dependent perception is robust against noise-vocoding (Jaekel et al., 2017). In fact, cochlear-implant users demonstrate similar if not stronger rate-dependent perception compared with individuals with normal hearing, corroborating that listener compensation against signal degradation maintains rate-dependent perception (Jaekel et al., 2017).

In addition to the observed rate effect under different conditions of signal degradation in both experiments, two additional findings warrant mentioning with regard to previous studies on rate-dependent perception and phonetic categorization more generally. Firstly, despite the fact that under signal degradation processing resources are likely taxed, the present results differ from previous studies testing rate-dependent perception in a foreign language or a dual task situation. For instance, Bosker et al. (2017) showed that if context sentences were presented under higher cognitive load, listeners reacted as if the context speech was fast, that is, they gave overall more long vowel responses on subsequent target categorization. Note that we would only predict a similar “shrinkage of time” effect in our Experiment 1, where the contexts were degraded but the targets were not. However, what we found was a main effect of noise versus quiet in Experiment 2 where it was not predicted to arise. Given this inconsistency and since in both experiments the factor Context was involved in further interactions (with Rate and/or Continuum) the present results do not speak to an account in which energetic-masking-induced cognitive load makes listeners miss samples in the speech signal, speeding up time perception.

Secondly, in addition to differences between context conditions in the magnitude of the rate effect (i.e., in Experiment 1), we found differences across conditions in the precision of perceiving the vowel duration continuum as indicated by the steepness of the categorization functions. With the exception of the noise condition in Experiment 1, the categorization of the vowel duration continuum was less precise in the degraded conditions than the clear condition. Note that in Experiment 1, where the targets were always presented in the clear, it is not entirely clear why the precision in perception of the vowel duration continuum following a reverberating context should be reduced. We speculated that the smearing of spectral information over time in reverberation likely reduces listeners’ reliance on temporal cues in general and hence affected the reliance on the actual vowel duration during target categorization. In Experiment 2, the most likely explanation of less precise target categorization is that also the targets were degraded and hence the actual vowel duration could not be assessed as accurately as in the clear.

The rate-dependent perception effect tested in the present study is an example of an acoustic context effect. It has also been referred to as “temporal contrast effect” (Bosker et al., 2020a) and is behaviorally very similar to “spectral contrast effects,” whereby the spectral characteristics of a context sentence (e.g., relatively high first formant (F1)) influences subsequent target perception (e.g., biasing perception of an /ɪ/–/ɛ/ F1 continuum towards /ɪ/; Sjerps et al., 2011; Stilp & Assgari, 2018). Even though both types of acoustic context effect are contrastive in nature and are typically tested using similar experimental designs, recent studies suggest they involve distinct processing mechanisms. For instance, while temporal contrast effects are immune to selective attention (Bosker et al., 2020a), spectral contrast effects are strongly modulated by selective attention (Bosker et al., 2020b; Feng & Oxenham, 2018). This raises the question whether the present modulation of rate-dependent perception by signal degradation of the context would generalize to spectral contrast effects. Reverberation would be an interesting form of degradation to test in this respect as it obscures the temporal characteristics of the context (as in Experiment 1), while actually “smearing out” stable spectral properties across time, thus perhaps even enhancing spectral contrast effects (Stilp et al., 2016).

The present outcomes also speak to the mechanisms of acoustic context perception proposed in Bosker et al. (2017). They put forward the idea that acoustic context effects, in both the temporal (tested here) and spectral domain, involve at least two processing stages: a first stage encompassing early and automatic perceptual normalization processes, while a second stage involves later cognitive adjustments, for instance driven by indexical speech properties (Reinisch, 2016b). We may speculate that the reduction of the rate effect by signal degradation observed here (i.e., perceptual load) arises at the first perceptual stage, while higher-level influences such as the perceived acceleration of time, induced by cognitive load, arise at a later stage. Eye-tracking experiments quantifying the time-course of different types of contexts relative to speech rate have started assessing the value of this idea (cf. Kaufeld et al., 2020; Maslowski et al., 2020; Reinisch & Sjerps, 2013).

The present experiments present a first step towards exploring the consequences of different types of listening environments on the functioning of low-level perceptual processes that listeners use during speech perception. Different results in the two experiments reveal the value of experimental control, while also advocating the use of more naturalistic auditory environments. That is, while Experiment 1 revealed some constraints on the temporal encoding of speech rate using artificial stimuli with sudden signal quality transitions, Experiment 2—in turn—demonstrated that listeners can adapt to challenging listening situations if those are stable within an utterance. Overall, we showed that listeners are able to maintain rate-dependent perception in noisy or reverberant conditions (in both experiments)—be it with small reductions of the effect depending on the precise experimental setting.

## Data Availability

The datasets generated and analyzed during the current study, as well as example stimuli are available in the Open Science Framework repository under a CC-By Attribution 4.0 International license (https://osf.io/4fgkz/). None of the experiments was preregistered.

## Footnotes

1 One could speculate whether participants recruited via the web are more variable in their behavior than participants in the lab. However, increased variability would have worked against finding differences between conditions. Note also that our dependent variable (phoneme categorization into long vs. short vowel) is not time sensitive and hence unlikely to be influenced by the lab-based versus web-based settings. Reinisch and Penney (2019) provide a direct comparison of results from a phoneme categorization task where participants were tested in the lab versus via the internet and show no difference. Even studies on time-sensitive variables suggest comparability of results across settings (e.g., `Gould et al., 2015; `Kim et al., 2019).

2 https://groups.linguistics.northwestern.edu/speech_comm_group/documents/praat%20scripts/MixSpeechNoise.praat (last accessed 23 March 2021).

3 The impulse response in the plugin refers to the “Bright big room” provided by EMES studio monitor systems http://www.emes.de/pageseng/products/Impulsresponse/eimpulse.htm (last accessed 22 May 2021). The properties in the respective documentation are the following: Reverberation time: 1.865 s, wet level −20 dB to −1dB, ErRefl.-time 0-80 ms, diffuse time 81-1865 ms.

4 Note that a direct comparison between experiments has to be taken with caution, since only in Experiment 1 participants responded to identical targets across conditions. However, the full cross-experiment model can be found on OSF. The only other interaction of factors with Experiment concerns the effect of Continuum comparing the clear versus noise conditions, which was smaller in Experiment 1.
